# Racial Disparities in Diabetes-related Preventive Care: Results From the Missouri Behavioral Risk Factor Surveillance System

**Published:** 2006-06-15

**Authors:** Joseph William LeMaster, Fungai Chanetsa, Julie M Kapp, Brian M Waterman

**Affiliations:** Department of Family and Community Medicine, University of Missouri–Columbia School of Medicine; Northwest Missouri State University, Maryville, Mo; Department of Health Management and Informatics, University of Missouri–Columbia, Columbia, Mo; Waterman Research Solutions, St Louis, Mo

## Abstract

**Introduction:**

Racial disparities exist in the rates of diabetes complications in the United States and in the state of Missouri. It is unclear to what degree such disparities involve diabetes-related preventive care. We sought evidence for racial disparities in diabetes-related preventive care between non-Hispanic blacks and whites in Missouri.

**Methods:**

We analyzed data from the Missouri Behavioral Risk Factor Surveillance System from 1994 through 2002. This state-specific survey is conducted annually among a representative sample of Missourians. We examined data from 842 Missourians who reported a diagnosis of type 1 or type 2 diabetes and who had consulted a health professional in the 12 months before they were interviewed. We analyzed reported receipt of glycosylated hemoglobin testing, foot examinations, and dilated eye examinations in the year before interview.

**Results:**

Non-Hispanic blacks were significantly less likely than whites to report having had glycosylated hemoglobin testing (odds ratio [OR], 0.47; 95% confidence interval [CI], 0.22–0.99) but more likely to report having received foot examinations (OR, 1.99; 95% CI, 1.21–2.39). There was no difference between blacks and whites in the probability of dilated eye examinations (OR, 1.49; 95% CI, 0.94–2.36).

**Conclusion:**

Compared with whites, non-Hispanic blacks in Missouri receive adequate screening for diabetic complications but not for glycemic control. Further studies are needed to investigate whether these disparities are linked to differences in the rate of diabetes complications in Missouri.

## Introduction

In the last decade, concerted efforts have been made to increase regular screening for diabetes complications to improve health outcomes. The American Diabetes Association recommends annual foot and dilated eye examinations; measurement of glycosylated hemoglobin (HbA1c), lipids, creatinine, cholesterol, and urine protein; and biannual measurement of blood pressure (1). These recommendations have led to considerable increases in the use of these tests in the last decade (2-4) and associated decreases in the rate of diabetes complications (2-6). Because provision of these diabetes preventive examinations is monitored by many large clinics (3,7,8), by the U.S. Department of Veterans Affairs (9), and by population-based surveys, including the Medicare Current Beneficiary Survey (10), the National Health and Nutrition Examination Survey (NHANES) (2), and the Behavioral Risk Factor Surveillance System (BRFSS) (11), these measures make it possible to compare diabetes quality of care across diverse populations and health care systems.

U.S. incidence rates for diabetes mellitus and complications from diabetes are higher for racial minority groups than for whites (10,12-20). Racial disparities in routine preventive diabetes care may contribute to these higher rates (10,12,13,21-24). Non-Hispanic black adults with diabetes who receive health care from U.S. managed care organizations are reportedly less likely than whites to receive annual HbA1c or eye screening (13,21). Reports from nationally representative U.S. population data are less consistent: one study analyzing Medicare claims data from more than 300,000 beneficiaries nationwide found that black Medicare beneficiaries are about a third less likely than whites to have HbA1c measured or to be referred for eye examinations (22). In contrast, reports analyzing data from Veterans Affairs facilities, the NHANES, and the BRFSS have not found racial differences in HbA1c testing, eye care, insulin use, or blood pressure treatment for individuals with diabetes (5,23,25). These findings suggest that some U.S. populations may experience racial disparities in diabetes care, whereas others do not.

In some U.S. states, large racial disparities exist in the rates of diabetes complications. From 1992 to 2002 in Missouri, diabetes-related deaths among non-Hispanic blacks occurred at twice the rate among whites, and emergency department visits for diabetes-related complications among blacks were about four times more frequent than among whites (26). Given that diabetes preventive care reduces diabetes-related complications and mortality, we hypothesized that diabetes preventive care would be less frequently received by non-Hispanic blacks than by whites in Missouri. We used state-specific data collected from the Missouri BRFSS from 1994 through 2002 to determine whether preventive diabetes care in Missouri differed by race and whether these differences changed over time.

## Methods

### Data source and study population 

The U.S. Centers for Disease Control and Prevention (CDC) collects annual, state-specific data for the BRFSS in all 50 states and the District of Columbia. The Missouri BRFSS uses a disproportionate stratified sampling methodology to select respondents from seven state health regions including the city of St Louis. Data are collected through telephone interviews among a sample of noninstitutionalized adults (aged 18 years and older) living in households with a telephone (27).

In this analysis, we included 842 Missourians who reported having a medical diagnosis of diabetes mellitus and non-Hispanic black or white race, who consulted a physician or health professional for a routine checkup in the year before being interviewed, and who answered questions about their diabetes preventive care during that period. Interviews were conducted in 1994, 1996, 1997, 2000, and 2002. We compiled data for these survey years to determine whether there was any trend in the frequency of reported examinations. We excluded other racial or ethnic groups that comprised only 4% of the total BRFSS sample because the small sample size precluded statistical analysis.

The BRFSS includes questions about preventive health practices and risk behaviors that are linked to chronic diseases, injuries, and preventable infectious diseases in the adult, noninstitutionalized civilian population. The set of questions varies somewhat among states in a given year and from year to year in a given state. Our analysis focused on a common set of questions about diabetes-related preventive care services (outcomes) that were reported in Missouri during the years we analyzed: whether respondents reported that, in the 12 months before being interviewed, a health professional had conducted a "hemoglobin A1c" blood test, checked their feet for any sores or irritations, or performed an eye examination in which the pupils were dilated. To focus our analysis on the quality of health care received by respondents with diabetes, we included only respondents who had been to a health professional in the 12 months before being interviewed. These data were gathered for the Missouri BRFSS in each of the survey years included in this study. Missouri BRFSS survey methods and wording of questions about the diabetes preventive care outcomes we analyzed did not change substantially during this period. For the years analyzed, the Missouri BRFSS included questions about cholesterol and blood pressure screening in 1997 only, so these questions were not included in our analysis.

### Data analysis

The BRFSS data are weighted by CDC to correct for the differences in probability of selection. The weighting takes into account the number of adults in the household, the number of telephones per household, and telephone coverage. In addition, poststratification weighting accounts for nonresponse by age, race, and sex and is based on the most recent census data in each U.S. state. This weighting allows inferences about the state population to be made from the sample (27). In all of our analyses, we adjusted and applied these weights to account for the sampling scheme in Missouri.

We conducted descriptive analyses initially using two-way contingency tables. We used multivariate logistic regression to estimate the association between race (non-Hispanic blacks compared with whites) and preventive care outcomes. We tested whether other respondent characteristics individually or jointly confounded these associations using likelihood ratio tests in these models. Characteristics were included in the final model for each outcome as adjustment variables if they changed the crude odds ratios (ORs) describing the effect of race on the preventive care outcome under consideration by more than 10%. We adjusted all models for income, employment, and enrollment in either a public or private health insurance plan. We also used likelihood ratio tests to assess interactions between race and other respondent characteristics (including the year respondents were interviewed). Item nonresponse was minimal except for income, which was not reported by 14% of respondents. We used linear regression to impute income when these data were missing. We replaced missing income values with those estimated by a multivariate linear regression model that estimated income as a function of all other variables that we included in the preventive care models. We then refit the final preventive care models using this imputed income variable. To determine the degree to which nonresponse by non-Hispanic black respondents may have led to selection bias in our analysis, we used a logistic regression model to estimate the association between race and nonresponse for each preventive care outcome that was asked by the BRFSS in the years we examined. We adjusted those estimates for sex, age, income, education, employment status, health insurance coverage, marital status, body mass index (BMI), and the year respondents were interviewed. We assessed model calibration for all models with the Hosmer-Lemeshow test (28). All data management and analyses were done with SAS, version 8.02 (SAS Institute Inc, Cary, NC). We used an α level of .05 to define significance. The study was approved by the University of Missouri Institutional Review Board.

## Results

Non-Hispanic blacks represented 16% of the Missouri population with diabetes who were sampled during the years we examined (Table 1). These respondents were younger, had slightly less income, and were less likely to be married than white respondents. Sex, education, BMI, health coverage, and employment did not differ by race.

Among the sample, 66% reported having had their feet checked by a health professional in the past year, with proportionally higher rates among non-Hispanic blacks (Table 2). Crude ORs indicated that blacks were more likely than whites to have had their feet checked in the past year. After adjustment for employment, enrollment in health insurance, BMI, and income, this difference remained significant (OR, 1.99; 95% confidence interval [CI], 1.21–2.39).

Sixty-seven percent of respondents reported having had a dilated eye examination in the past year, with a higher proportion reporting this outcome among blacks (72%) than whites (68%) (Table 2). Although the crude OR indicated that blacks were more likely to have had an eye examination than whites, this difference was not statistically significant. After the model was adjusted for health insurance coverage, income, age, and employment, the increased OR for blacks receiving an eye examination remained nonsignificant (OR, 1.49; 95% CI, 0.94–2.36).

Among the sample, 90% reported having had at least one HbA1c test in the past year. Blacks were less likely to report having received this test than whites in the crude analysis (Table 2). After adjustment for health insurance coverage, employment, and income, this difference remained significant (OR, 0.47; 95% CI, 0.22–0.99).

There was no evidence that blacks were selectively excluded from the models because of nonresponse (Table 3). After the model was adjusted for respondent characteristics and the year of interview, non-Hispanic blacks were more likely to respond to questions about foot examination, whereas there was no difference in nonresponse between whites and blacks for the eye examination and HbA1c models.

There was no evidence of interaction between race and any of the adjustment variables in the regression models. There was also no evidence for increasing or decreasing trend by year for any of the diabetes preventive care outcomes that we considered (data not shown). All statistical models described revealed appropriate (i.e., nonsignificant) calibration based on the Hosmer-Lemeshow test.

## Discussion

This study suggests that there was substantial underuse of a key test of diabetes control, HbA1c level, among non-Hispanic black Missourians with diabetes from 1994 to 2002. Blacks reported adequate preventive care for at least two potential diabetes complications, retinopathy and neuropathy, at least as frequently as whites; foot examination was reported more frequently by blacks, and eye care did not differ by race. Our finding of a disparity in HbA1c testing differs from a previous national BRFSS analysis, which found that non-Hispanic blacks did not differ from other groups in receipt of preventive services (5).

Our results cannot be explained by differences in access to medical care between blacks and whites, because all respondents had been examined by a physician in the last year. We included in our analysis only respondents who had consulted a health care professional, because the BRFSS does not include questions about why respondents thought they did or did not have access to health care professionals. Although people with diabetes may not receive diabetes preventive care — either because they do not have access to a care provider or because the provider does not deliver the care — an analysis exploring reasons why individual respondents did not consult a health care professional was beyond the scope of this study.

Low-income patients and patients without health insurance are less likely to be up-to-date on preventive services of all types (29); however, all of our regression models adjusted for employment, income, and enrollment in health insurance. Other factors related to the health care encounter may also have an influence; U.S. physicians are under pressure to see more patients in less time (24), a situation that reduces time spent on diabetes preventive care (30). In one survey study of physicians, they reported that their biggest challenge to adequate treatment of diabetes was not patients' race; it was inadequate time and resources (31).

Differences in patients' and health care providers' perceptions about the importance of diabetes in the patient's life may strongly influence providers' delivery of preventive diabetes care and patients' achievement of optimal glycemic control (31,32). Physicians in one large U.S. health maintenance organization stated that there seemed little reason to order HbA1c tests for black patients because these tests had been consistently high in the recent past (i.e., reflecting poor glycemic control) (21). Similar practices among Missouri physicians may have contributed to our finding that blacks reported having had fewer HbA1c tests than whites in that state. In contrast, blacks may have reported that they had received foot and eye examinations at least as frequently  as whites because both providers and patients perceived correctly that diabetes foot and eye complications occur frequently among blacks (and thus are important to detect early). The alternative explanation, that black respondents preferentially remembered foot examinations better and HbA1c tests worse than white respondents, seems less reasonable.

Black patients depend heavily on the medical system to help them manage their diabetes as they grow older because they realize that their risk of a grave complication is high (33). Non-Hispanic black, Hispanic, and white patients all attend ophthalmologic, podiatric, and weight reduction clinics equally when referred by their primary care physician (21). Although black diabetes patients make fewer outpatient physician visits and use emergency services more than whites (10), we are unaware of any published evidence that suggests that they are indifferent about receiving diabetes preventive care.

Given the relatively high baseline prevalence of the outcomes examined in this study, the adjusted ORs we report, although valid and sound measures of association, may not approximate relative risk to the extent that they would if the baseline prevalence of the outcomes was low. Although other methods of presenting findings are available (i.e., model-adjusted probabilities), we chose to report model findings in the more common format of model-adjusted ORs.

This study has several limitations. First, results from the BRFSS may not equally represent all black residents in Missouri. The BRFSS does not collect data on people without telephones. Although our adjusted BRFSS weights accounted for this at the state level, blacks from rural areas of Missouri, where telephones are less common than in urban areas, may have been underrepresented (27). On the other hand, our analysis may have overestimated preventive service use because individuals who cannot afford a telephone are also less likely to receive regular diabetes preventive care (34). Second, the BRFSS elicits responses about medical care from patients rather than from health care providers. Self-reported data are subject to recall bias. Differences in socioeconomic status or cultural or language barriers may have differentially affected reporting patterns for blacks compared with whites. Third, because the data from respondents from other ethnic groups were sparse, our analysis only compared non-Hispanic blacks to whites; however, similar observations have been made about the difference in quality of care between Hispanics and non-Hispanics. In fact, Hispanics have less frequent HbA1c testing than non-Hispanic blacks (21). Fourth, numerous responses to the HbA1c question were missing, making the confidence intervals for that outcome quite wide. Fifth, nonresponse to diabetes preventive care questions was high for the HbA1c test model; however, we found no association between race and nonresponse for that outcome. Sixth, to examine trends in diabetes care over time, we limited the number of outcomes that we analyzed to those that were repeatedly included in the Missouri BRFSS during the years we studied. Although we cannot generalize these results to other preventive care practices, these findings are consistent with what others have found (10,12,13,17,21,35,36). Finally, because the BRFSS only surveys noninstitutionalized people, our results cannot be generalized to people who live in nursing homes, prisons, or other institutions.

We cannot conclude, based on our results, that disparities in HbA1c testing lead to differences in rates of diabetes-related complications and mortality in Missouri, because the BRFSS does not collect information on the occurrence of diabetes complications; however, the results are suggestive. In Michigan, non-Hispanic blacks with diabetes in a health maintenance organization had higher HbA1c values and poorer renal function than whites, and blacks did not receive recommended levels of preventive diabetes care (37). In another Missouri survey, black youths with diabetes had substantially poorer glycemic control than their white counterparts, although that study did not investigate the association between glycemic control and the quality of medical care that respondents received (38). Another recent survey of non-Hispanic blacks in Missouri revealed that respondents did not feel that they received the same quality of health care as whites (26). Further studies should investigate the association between racial disparities in diabetes preventive care and the occurrence of complications from diabetes in midwestern U.S. states.

## Figures and Tables

**Table 1:** Demographic Characteristics of Respondents With Diabetes, Missouri Behavioral Risk Factor Surveillance System, 1994–2002

**Characteristic**	**Non-Hispanic Black**	**White**	**All**	** *P* Value^a^**
**No. in sample** (% of state population, weighted^b^)	101 (16)	741 (84)	842	NA
**Mean age, y (SD)**	57.0 (16.2)	61.2 (12.7)	61.1 (13.3)	.04
**Annual household income, %**				
<$10,000	20	12	13	.07
$10,000-19,999	22	27	26	
≥$20,000	58	61	61	
**Marital status, %**				
Married	44	65	62	.003
Not married	56	35	38	
**Sex, %**				
Female	47	48	51	.45
Male	53	52	49	
**Education, %**				
Some high school	21	20	20	.56
High school graduate	31	40	39	
Some college	30	23	24	
College graduate	18	17	17	
**Body mass index, %**				
Normal (<25)	30	22	24	.23
Overweight (25.0-29.9)	45	43	43	
Obese (≥30)	25	35	33	
**Have insurance, %**				
Yes	87	93	92	.11
No	13	7	8	
**Currently employed, %**				
Yes	40	31	33	.20
No	60	69	67	

NA indicates not applicable.

a *P *values determined by chi-square test.

b Weighted to U.S. census data for Missouri.

**Table 2:** Likelihood of Non-Hispanic Blacks Having Received Diabetes Preventive Care Compared With Whites, by Type of Care, Missouri Behavioral Risk Factor Surveillance System, 1994–2004^a^

**Preventive Care Service (n in Final Model)**	**Respondents Examined, %**	**Non-Hispanic Black, No. (%)**	**White, No. (%)**	**Non-Hispanic Blacks Compared With Whites**		
				**Crude OR (95% CI)**	**Adjusted^b^ OR (95% CI)**	**Adjusted^b^ OR, Imputed^c^ (95% CI)**
Foot examination (675)	66	87 (78)	373 (66)	1.80 (1.12-2.90)	2.13 (1.26-3.72)	1.99 (1.21-2.39)
Dilated eye examination (739)	67	85 (72)	424 (68)	1.22 (0.79-1.89)	1.65 (1.02-2.69)	1.49 (0.94-2.36)
HbA1c test (530)	90	76 (87)	415 (94)	0.41 (0.20-0.85)	0.46 (0.22-0.99)	0.47 (0.22-0.99)

OR indicates odds ratio; CI, confidence interval; HbA1c, hemoglobin A1c.

a Numbers and percentages represent weighted observations.

b Foot examination model adjusted for employment, health insurance coverage, income, and body mass index; eye examination model adjusted for employment, health insurance coverage, income, and age; HbA1c test model adjusted for employment, health insurance coverage, and income.

c Missing values for reported income were estimated using a multivariate linear regression model.

**Table 3:** Association Between Race and Nonresponse to Questions About Diabetes Preventive Care Among Non-Hispanic Black and White Respondents With Diabetes (N = 800), Missouri Behavioral Risk Factor Surveillance System, 1994–2004

**Preventive Care Service**	**Total Missing Responses, No. (%)**	** Missing Responses Among Non-Hispanic Blacks, No. (%)**	**Missing Responses Among Whites, No. (%)**	**Nonresponse for Non-Hispanic Blacks Compared With Whites**	
				**Crude OR (95% CI)**	**Adjusted^a^ OR (95% CI)**
Foot examination	76 (9)	8 (8)	68 (9)	0.40 (0.15-1.06)	0.22 (0.08-0.61)
Dilated eye examination	16 (2)	1 (1)	15 (2)	0.31 (0.04-2.42)	0.33 (0.03-3.6)
HbA1c test	262 (31)	38 (37)	224 (30)	0.77 (0.45-1.32)	0.82 (0.41-1.64)

OR indicates odds ratio; CI, confidence interval; HbA1c, hemoglobin A1c.

a Models were adjusted for sex, age, income, education, employment status, health insurance coverage, marital status, body mass index, and year respondents were interviewed.
